# Getting Chilly

**Published:** 1866-12

**Authors:** 


### GETTING CHILLY.

The reader has no doubt observed many times that if in very severe winter weather he remains in the house several days, the body gets very chilly; while you are warming the feet and hands before the fire, the cold chills run down the back; or if you go even from the fire to the window to look upon the snow, disagreeable creeps run all over the body; and whether in these, or under any other circumstances, persons have an unpleasant chilliness, it is the result of a sluggish circulation and an imperfect digestion—so little life-giving air is breathed, and so little exercise is taken that the nutriment is not drawn from the food eaten, the blood grows poor, and lifeless, and cold; loses its heating power, and the body begins to freeze and die. But let a few hours be spent in the cool, out-door air in some exhilerating employment or pastime, and there is an entire change in the whole physical and mental condition; the fire of life kindles in the eye, smiles light up the face, and the man is himself again.

